# Synthesis and Antifungal Screening of 2-{[1-(5-Alkyl/arylalkylpyrazin-2-yl)ethylidene]hydrazono}-1,3-thiazolidin-4-ones †

**DOI:** 10.3390/molecules21111592

**Published:** 2016-11-23

**Authors:** Veronika Opletalova, Jan Dolezel, Jiri Kunes, Vladimir Buchta, Marcela Vejsova, Marta Kucerova-Chlupacova

**Affiliations:** 1 Department of Pharmaceutical Chemistry and Pharmaceutical Analysis, Faculty of Pharmacy in Hradec Kralove, Charles University, Heyrovskeho 1203, 500 05 Hradec Kralove, Czech Republic; opletalova@faf.cuni.cz; 2 GlaxoSmithKline, Hvezdova 1734/2c, 140 00 Prague, Czech Republic; jan.j.dolezel@gsk.com; 3 Department of Inorganic and Organic Chemistry, Faculty of Pharmacy in Hradec Kralove, Charles University, Heyrovskeho 1203, 500 05 Hradec Kralove, Czech Republic; kunes@faf.cuni.cz; 4 Department of Biological and Medical Sciences, Faculty of Pharmacy in Hradec Kralove, Charles University, Heyrovskeho 1203, 500 05 Hradec Kralove, Czech Republic; buchta@faf.cuni.cz (V.B.); vejsova@faf.cuni.cz (M.V.); 5 Department of Clinical Microbiology, University Hospital Hradec Kralove, Sokolska 581, 500 05 Hradec Kralove, Czech Republic

**Keywords:** acetylpyrazine, thiosemicarbazones, 1,3-thiazolidin-4-ones, antifungal, *Candida glabrata*

## Abstract

Two novel thiosemicarbazones and eight novel 2-{[1-(5-alkyl/arylalkylpyrazin-2-yl)ethylidene]hydrazono}-1,3-thiazolidin-4-ones were prepared and tested against a panel of eight fungal strains–*Candida albicans* ATCC 44859, *Candida tropicalis* 156, *Candida krusei* E 28, *Candida glabrata* 20/I, *Trichosporon asahii* 1188, *Aspergillus fumigatus* 231, *Lichtheimia corymbifera* 272, and *Trichophyton interdigitale* 445. 1,3-Thiazolidin-4-ones exhibited activity against all strains, the most potent derivative was 2-{[1-(5-butylpyrazin-2-yl)ethylidene]hydrazono}e-1,3-thiazolidin-4-one. Susceptibility of *C. glabrata* to the studied 1,3-thiazolidin-4-ones (minimum inhibitory concentrations (MICs) were in the range 0.57 to 2.78 mg/L) is of great interest as this opportunistic pathogen is poorly susceptible to azoles and becomes resistant to echinocandins. Antifungal potency of thiosemicarbazones was slightly lower than that of 1,3-thiazolidin-4-ones.

## 1. Introduction

Fungal infections, especially invasive ones, represent a serious problem. Whilst topical fungal diseases are quite common and cause considerable morbidity, they are generally not life-threatening [1]. On the contrary, it has been estimated that invasive fungal infections are responsible for the deaths of 1.5 million people each year [2]. The increased incidence of life threatening systemic fungal infections is mainly due to the increasing numbers of immunocompromised people [2,3]. Besides, infections that were once uncommon emerge more frequently in the United States and Europe as a result of international travel, immigration from endemic areas, and changing climate conditions [4]. Fungal resistance also prevents successful treatment of mycoses [5,6,7,8]. Therefore, searching for new drugs and therapeutic options is of high importance [9,10,11].

**Figure 1:**
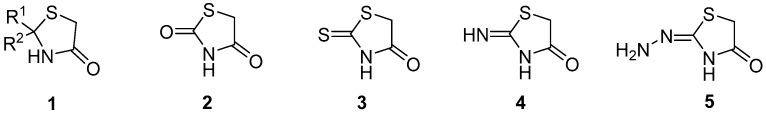
Structures of 2-substituted derivatives of 1,3-thiazolidin-4-one.

1,3-Thiazolidin-4-ones are very versatile compounds both as synthetic intermediates and potential drugs [12,13,14,15,16,17,18,19]. Substituents in the 2-, 3-, and 5-positions of the basic skeleton may be modified. According to substitution in the 2-position, 1,3-thiazolidin-4-one derivatives can be subdivided into several classes: alkyl or (hetero)aryl substituted thiazolidin-4-ones **1**, thiazolidine-2,4-diones **2**, rhodanines **3**, 2-iminothiazolidin-4-ones (pseudohydantoins) **4**, and 2-hydrazonothiazolidin-4-ones **5** (Figure 1). All of them can be further substituted on N-3 and/or methylene group in position 5 of the 1,3-thiazolidin-4-one skeleton and compounds **4** and **5** also on imino or amino group of the substituent [12].

**Figure 2:**
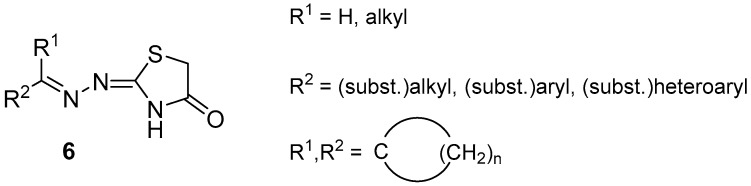
General formula of 2-hydrazonothiazolidin-4-ones derived from aldehydes and ketones

Many 2-hydrazono-1,3-thiazolidin-4-ones of general formula **6** (Figure 2) have been reported in the literature. One of the synthetic approaches to these compounds consists in the reaction of the corresponding thiosemicarbazones with α-halogenoalkanoic acids or their derivatives [12,14,15,17,18,19].

**Scheme 1:**
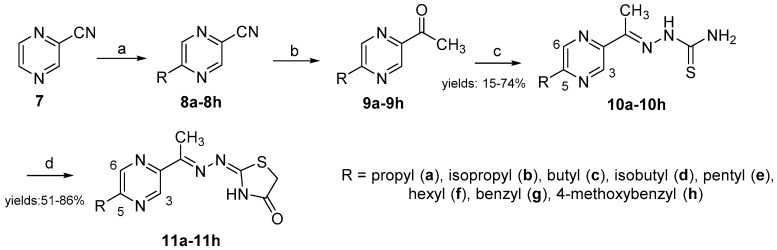
Synthesis of the compounds **11a**–**11h**. *Reagents and conditions*: (a) (aryl)alkanoic acid, AgNO_3_, (NH_4_)_2_S_2_O_8_, water, 80 °C; (b) CH_3_MgI, Et_2_O; (c) thiosemicarbazide, MeOH, CH_3_COOH; (d) α-chloroacetic acid, anhydrous EtOH, sodium acetate.

A series of thiosemicarbazones **10a**–**10f** has previously been prepared in our laboratory using the procedure shown in Scheme 1. Thiosemicarbazones **10a**–**10f** have already been tested for antifungal, antitumor, and iron-chelating properties [20]. Their antimycobacterial activity has been reported as well [21]. Thiosemicarbazones **10g** and **10h** were prepared later and are novel compounds.

In the present paper, we report on the synthesis and antifungal properties of 2-{[1-(5-alkyl/arylalkylpyrazin-2-yl)ethylidene]hydrazono}-1,3-thiazolidin-4-ones **11a**–**11h** obtained from thiosemicarbazones **10a**–**10h** by cyclization with α-chloroacetic acid (Scheme 1). Antifungal effects of the new thiosemicarbazones **10g** and **10h** will be reported as well.

## 2. Results and Discussion

The paper is a follow-up of our long-term research activities aimed at studies of pyrazine derivatives. Most of these studies were devoted to derivatives of pyrazinecarboxylic acid since pyrazinamide is a well-known antimycobacterial drug [22,23,24,25,26,27,28,29,30]. Ring substituted acetylpyrazines have also been studied, but to a lesser extent. Pyrazine is an electron-deficient nitrogen heterocyclic base which does not undergo Friedel-Crafts acylations. Various acetylpyrazines have been obtained using homolytic acetylation [31]. However, homolytic reactions often yield a mixture of monoacylated, diacetylated and other derivatives, and the isolation of the desired product in a sufficient amount is sometimes rather difficult [32,33,34,35,36]. Thus, homolytic acetylation of 2,5-dimethylpyrazine gave 2,5-diacetyl-3,6-dimethylpyrazine and 2-acetyl-3,5,6-trimethylpyrazine, but not 2-acetyl-3,6-dimethylpyrazine [33]. Homolytic acetylation of pyrazine-2-carbonitrile yielded 5-acetylpyrazine-2-carbonitrile as expected [37], but when we tried to apply the same reaction conditions to alkylated pyrazine-2-carbonitrile, we were not able to get a pure product from the reaction mixture. 5-Acetylpyrazine-2-carbonitrile was then used for the preparation of (*E*)-2-[1-(5-cyanopyrazin-2-yl)ethylidene]hydrazinecarbothioamide. This thiosemicarbazone exhibited neither antifungal nor antiproliferative activity [20]. Therefore, only 5-alkylated acetylpyrazines have been further used in our studies concerning their derivatives, such as chalcones [38,39] and compounds reported in the present study.

Antifungal properties of compounds containing sulfur and nitrogen have been well documented in literature [40,41,42,43,44,45,46]. Among these substances also various thiosemicarbazones [47,48,49,50,51,52] and derivatives of 1,3-thiazolidin-4-one were reported [53,54,55,56,57,58,59,60,61,62,63]. Compounds of this type were also studied by our research group and the results are discussed below.

### 2.1. Chemistry

Thiosemicarbazones **10g** and **10h** were prepared using the method reported in our previous paper [20]. Their spectral characteristics corresponded to the spectra of their previously reported congeners **10a**–**10f**. Hence, it could be concluded that they are also *E*-isomers. Details concerning the determination of the configuration on the double bond can be found in ref. [20].

All available thiosemicarbazones were then reacted with α-chloroacetic acid yielding 2-{[1-(5-alkyl/arylalkylpyrazin-2-yl)ethylidene]hydrazono}-1,3-thiazolidin-4-ones **11a**–**11h**. A slightly modified method of Hozien [64] was used. The products were characterized by melting points, IR, and NMR spectra and their purity was checked by thin layer chromatography (TLC) and elemental analysis.

### 2.2. Biology

The in vitro antifungal activity of all compounds was evaluated by the modified microdilution broth Clinical and Laboratory Standards Institute (CLSI) standards [65,66]. The organisms examined included *Candida albicans* ATCC 44859 (American Type Culture Collection, Manassas, VA, USA), *Candida tropicalis* 156, *Candida krusei* E 28, *Candida glabrata* 20/I, *Trichosporon asahii* 1188, *Aspergillus fumigatus* 231, *Lichtheimia corymbifera* (formerly *Absidia corymbifera*) 272, and *Trichophyton interdigitale* (formerly *T. mentagrophytes*) 445. All strains tested, except ATCC, were clinical isolates obtained from the Department of Clinical Microbiology, University Hospital and Faculty of Medicine, Charles University, Prague, Czech Republic. Comparison of the minimal inhibition concentrations (MICs) of thiosemicarbazones **10a**–**10f** reported previously [20] and novel thiosemicarbazones **10g** and **10h** with MICs of 2-{[1-(5-alkyl/arylalkylpyrazin-2-yl)ethylidene]hydrazono}-1,3-thiazolidin-4-ones **11a**–**11h** showed that antifungal activity of compounds **11a**–**11h** was slightly better than that of thiosemicarbazones **10a**–**10h**.

1,3-Thiazolidin-4-ones **11a**–**11h** were active to almost all fungal strains. Derivatives with medium length alkyl chains **11a** (propyl), **11c** (butyl), and **11e** (pentyl) were the most potent ones. This in good agreement with our previous studies of chalcones [38,67,68] and thiosemicarbazones [20], that also indicated that compounds with non-branched alkyls mostly exhibited better antifungal potency than their analogs with branched alkyls. Surprisingly, 2-{[1-(5-hexylpyrazin-2-yl)ethylidene]hydrazono}-1,3-thiazolidin-4-one (**11f**) was less potent than the corresponding thiosemicarbazone **10f** [20]. This clearly shows that the optimal length of alkyl substituent in the pyrazine ring may be different for various types of compounds. For the 1,3-thiazolidin-4-ones presented in this paper, the optimal length is four carbons (see butyl derivative **11c** in Table 1).

As can be seen in Table 1, MICs of fluconazole and voriconazole were uncommonly high for *C. tropicalis*. This indicates that the strain could have developed a resistance to azole antifungal agents during long-term passaging. However, as the most potent compounds (**11a**, **11c**, and **11e**) exhibited activity against the resistant strain, it can be presumed that their mechanism of action and/or resistance is different from that of azoles, and their antifungal activity is independent of the susceptibility of a given strain of *C. tropicalis* to azole derivatives.

As it was already mentioned in the introduction, invasive fungal infections, especially those caused by resistant pathogens, represent a serious health problem [5,6,7,69,70,71]. In immunocompromised patients, they have high mortality rates (20%–40% for *Candida albicans*, 20%–70% for *Cryptococus neoformans*, and 50%–90% for *Aspergillus fumigatus*) [72]. Moreover, new infections due to opportunistic fungi, have emerged recently [73,74,75,76,77]. One of these difficult to treat pathogens is *Candida glabrata*. It exhibits some special features and is more similar to *Saccharomyces cerevisiae* than to *Candida albicans* [78,79]. *C. glabrata* belongs to the main fungal opportunistic pathogen in humans. It is poorly susceptible to azole antimycotic agents [78] and becomes resistant to echinocandins [79,80,81]. Strain resistance to amphotericin B was also reported [82]. 2-{[1-(5-Alkyl/arylalkylpyrazin-2-yl)ethylidene]hydrazono}-1,3-thiazolidin-4-ones **11a**–**11h** presented here showed promising activity against *C. glabrata*. The most potent derivative, **11c** exhibited good activity against all studied fungal pathogens. These data make them prospective antifungal agents that deserve further studies.

## 3. Materials and Methods

### 3.1. Chemistry

Pyrazine-2-carbonitrile (Sigma-Aldrich, Prague, Czech Republic) was used as a starting compound. It was alkylated to yield intermediates **8a**–**8h**, and these in turn were converted to the corresponding acetylpyrazines **9a**–**9h** using methods reported previously [83,84]. Thiosemicarbazones **10a**–**10f** were prepared and characterized in our previous paper [20]. Thiosemicarbazones **10g** and **10h** were prepared analogously using commercially available analytical grade thiosemicarbazide (Lachema, Brno, Czech Republic). Commercially available pure chloroacetic acid (Sigma-Aldrich, Prague, Czech Republic) and pure crystalline sodium acetate (Lachema, Brno, Czech Republic) were used for the cyclization of thiosemicarbazones. The purity of the products was checked by thin layer chromatography on TLC aluminium sheets, silica gel 60 F_254_ (Merck, Darmstadt, Germany); mixtures of light petroleum and ethyl acetate 80:20 and 60:40 were used as mobile phases. Analytical samples were dried over anhydrous phosphorus pentoxide under reduced pressure at room temperature. Melting points were determined on a Boëtius BHMK 73/4615 apparatus (VEB Analytik, Dresden, Germany) and are uncorrected. Elemental analyses (EA) were performed on an EA 1110 CHNS instrument (CE Instruments, Milano, Italy). IR spectra were recorded by the attenuated total reflection (ATR-Ge) method on Nicolet Impact 400 spectrometer or Nicolet 6700 IR spectrophotometer (Nicolet–Thermo Scientific, Madison, WI, USA). Characteristic wavenumbers are given in cm^−1^. ^1^H and ^13^C-NMR spectra were recorded at ambient temperature on a Varian Mercury-Vx BB 300 spectrometer (Varian Corp., Palo Alto, CA, USA) operating at 300 MHz for ^1^H and 75 MHz for ^13^C or VNMR S500 (Varian) spectrometer operating at 500 MHz for ^1^H-NMR and 125 MHz for ^13^C-NMR. Chemical shifts were recorded as δ values in ppm, and were indirectly referenced to tetramethylsilane (TMS) via the solvent signal (2.49 for ^1^H, 39.7 for ^13^C in DMSO-*d*_6_). Signal multiplicities are described as s, singlet; bs, broad singlet; m, multiplet; d, doublet and t, triplet. 

#### 3.1.1. General Procedure for the Preparation of Thiosemicarbazones **10a**–**10h**

5-Alkylated acetylpyrazine (0.01 mol) and thiosemicarbazide (0.01 mol) were dissolved in methanol (10–15 mL). Three drops of concentrated acetic acid were added, and the mixture was heated at reflux for 5 h. Then it was cooled, the product was removed by filtration and crystallized from ethanol. Thiosemicarbazones **10a**–**10f** (characterized in reference [20]) and two novel thiosemicarbazones **10g** and **10h** were prepared by this procedure.

*(E)-2-[1-(5-Benzylpyrazin-2-yl)ethylidene]hydrazinecarbothioamide* (**10g**): White solid; yield 42%; m.p. 209–214 °C; IR (ATR-Ge): 3382, 3147 (NH), 2914 (CH), 1613 (C=N) cm^−1^; ^1^H-NMR (500 MHz, DMSO-*d*_6_): δ 10.43 (1H, s, NH), 9.50 (1H, s, H-3), 8.56 (1H, s, H-6), 8.42 (1H, bs, NH_2_), 8.23 (1H, bs, NH_2_), 7.31–7.17 (5H, m, benzyl H-2, H-3, H-4, H-5, H-6), 4.15 (2H, s, CH_2_), 2.33 (3H, s, CH_3_); ^13^C-NMR (125 MHz, DMSO-*d*_6_): δ 179.3, 155.4, 148.1, 146.5, 142.6, 142.4, 139.0, 129.1, 128.7, 126.5, 40.7, 12.1; EA calculated for C_14_H_15_N_5_S (285.37): 58.92% C; 5.30% H; 24.54% N; 11.24% S. Found 58.61% C; 5.45% H; 25.08% N; 11.66% S.

*(E)-2-{1-[5-(4-Methoxybenzyl)pyrazin-2-yl]ethylidene}hydrazinecarbothioamide* (**10h**): White solid; yield 15%; m.p. 185–187 °C; IR (ATR-Ge): 3227, 3149 (NH), 2835 (CH), 1600 (C=N) cm^−1^; ^1^H-NMR (500 MHz, DMSO-*d*_6_): δ 10.42 (1H, s, NH), 9.49 (1H, d, *J* = 1.3 Hz, H-3), 8.52 (1H, d, *J* = 1.3 Hz, H-6), 8.42 (1H, bs, NH_2_), 8.23 (1H, bs, NH_2_), 7.23–7.16 (2H, m, AA′, BB′, benzyl H-2, H-6), 6.87–6.81 (2H, m, AA′, BB′, benzyl H-3, H-5), 4.07 (2H, s, CH_2_), 3.69 (3H, s, OCH_3_), 2.33 (3H, s, CH_3_); ^13^C-NMR (125 MHz, DMSO-*d*_6_): δ 179.3, 158.0, 155.8, 148.0, 146.5, 142.5, 142.3, 130.9, 130.1, 114.1, 55.2, 39.9, 12,1; EA calculated for C_15_H_17_N_5_OS (315.39): 57.12% C; 5.43% H; 22.21% N; 10.17% S. Found 56.76% C; 5.49% H; 21.99% N; 10.53% S.

#### 3.1.2. General Procedure for the Cyclization of Thiosemicarbazones **10a**–**10h** to 1,3-Thiazolidin-4-ones **11a**–**11h**

Thiosemicarbazone (7 mmol) and chloroacetic acid (0.99 g, 10.5 mmol) were dissolved in a minimum amount of anhydrous ethanol under stirring and heating to reflux. Then, 1.5% (*w/w*) ethanolic solution of sodium acetate (8 mL) was added, and the reaction mixture was heated under reflux for 10 h. After cooling, the precipitated crystals were sucked off, washed with water and 50 mL of water–ethanol mixture (1:1, *v/v*). Analytically pure products were obtained by crystallization from anhydrous ethanol. Using this procedure the following compounds were prepared:

*2-{[1-(5-Propylpyrazin-2-yl)ethylidene]hydrazono}-1,3-thiazolidin-4-one* (**11a**): Yellow solid; yield 51%; m.p. 216–217 °C; IR (ATR-Ge): 3126, 3016 (NH), 2961, 2871 (CH), 1702 (C=O), 1628 (C=N) cm^−1^; ^1^H-NMR (300 MHz, DMSO-*d*_6_): δ 12.11 (1H, bs, NH), 9.10 (1H, d, *J* = 1.5 Hz, H-3), 8.54 (1H, d, *J* = 1.5 Hz, H-6); 3.89 (2H, s, SCH_2_); 2.76 (2H, t, *J* = 7.5 Hz, CH_2_); 2.37 (3H, s, CH_3_), 1.78–1.61 (2H, m, CH_2_), 0.90 (3H, t, *J* = 7.5 Hz, CH_3_); ^13^C-NMR (75 MHz, DMSO-*d*_6_): δ 174.2, 166.7, 160.1, 157.3, 148.2, 143.0, 141.5, 36.6, 36.5, 33.2, 22.2, 13.7; EA calculated for C_12_H_15_N_5_OS (277.35): 51.97% C; 5.45% H; 25.25% N; 11.56% S. Found 51.58%; 5.49% H; 25.08% N; 11.92% S.

*2-{[1-(5-Isopropylpyrazin-2-yl)ethylidene]hydrazono}-1,3-thiazolidin-4-one* (**11b**). White solid; yield 49%; m.p. 225–226 °C; IR (ATR-Ge): 3125, 3082 (NH), 2964, 2922, 2871, 2826 (CH), 1704 (C=O), 1627 (C=N); ^1^H-NMR (300 MHz, DMSO-*d*_6_): 12.11 (1H, bs, NH), 9.10 (1H, d, *J* = 1.5 Hz, H-3), 8.58 (1H, d, *J* = 1.5 Hz, H-6), 3.90 (2H, s, SCH_2_), 3.21–3.04 (1H, m, CH), 2.38 (3H, s, CH_3_), 1.26 (6H, d, *J* = 6.9 Hz, CH_3_); ^13^C-NMR (125 MHz, DMSO-*d*_6_): 174.4, 166.8, 162.1, 160.4, 148.7, 141.9, 141.6, 33.5, 33.4, 22.4, 13.7; EA for C_12_H_15_N_5_OS (277.35) calculated 51.97% C, 5.45% H, 25.25% N, 11.56% S, found 51.64% C, 5.49% H, 25.15% N, 11.55% S.

*2-{[1-(5-Butylpyrazin-2-yl)ethylidene]hydrazono}-1,3-thiazolidin-4-one* (**11c**): Yellow solid; yield 42%; m.p. 210–212 °C; IR (ATR-Ge): 3066, 3016 (NH), 2955, 2929, 2869, 2779 (CH), 1718 (C=O), 1624 (C=N) cm^−1^; ^1^H-NMR (300 MHz, DMSO-*d*_6_): δ 12.11 (1H, bs, NH), 9.09 (1H, d, *J* = 1.4 Hz, H-3), 8.54 (1H, d, *J* = 1.4 Hz, H-6), 3.89 (2H, s, SCH_2_), 2.78 (2H, t, *J* = 7.4 Hz, CH_2_), 2.37 (3H, s, CH_3_), 1.73–1.58 (2H, m, CH_2_), 1.38–1.22 (2H, m, CH_2_), 0.88 (3H, t, *J* = 7.4 Hz, CH_3_); ^13^C-NMR (75 MHz, DMSO-*d_6_*): δ 174.2, 166.7, 160.1, 157.5, 148.2, 142.9, 141.5, 34.2, 33.2, 31.0, 22.0, 13.9, 13,5; EA calculated for C_13_H_17_N_5_OS (291.37): 53.59% C; 5.88% H; 24.04% N; 11.00% S. Found 52.70% C; 5.61% H; 24.00% N; 9.68% S.

*2-{[1-(5-Isobutylpyrazin-2-yl)ethylidene]hydrazono}-1,3-thiazolidin-4-one* (**11d**): Yellow solid; yield 40%; m.p. 202–204 °C; IR (ATR): 3134, 3070 (NH), 2953, 2927, 2868, 2798 (CH), 1705 (C=O), 1620 (C=N) cm^−1^; ^1^H-NMR (300 MHz, DMSO-*d*_6_): δ 12.11 (1H, bs, NH), 9.12 (1H, d, *J* = 1.4 Hz, H3), 8.52 (1H, d, *J* = 1.4 Hz, H6), 3.90 (2H, s, SCH_2_), 2.66 (2H, d, *J* = 6.9 Hz, CH_2_), 2.38 (3H, s, CH_3_), 2.15–1.97 (1H, m, CH), 0.88 (6H, d, *J* = 6.9 Hz, CH_3_); ^13^C-NMR (75 MHz, DMSO-*d*_6_): δ 174.2, 166.6, 160.1, 156.6, 148.2, 143.4, 141.5, 43.5, 33.2, 28.6, 22.3, 13.4; EA calculated for C_13_H_17_N_5_OS (291.37): 53.59% C; 5.88% H; 24.04% N; 11.00% S. Found 53.04% C; 5.79% H; 24.34% N; 10.37% S.

*2-{[1-(5-Pentylpyrazin-2-yl)ethylidene]hydrazono}-1,3-thiazolidin-4-one* (**11e**): Yellow solid; yield 57%; m.p. 181–182 °C; IR (ATR-Ge): 3083 (NH), 2955, 2862, 2791 (CH), 1719 (C=O), 1625 (C=N) cm^−1^; ^1^H-NMR (300 MHz, DMSO-*d*_6_): δ 12.11 (1H, bs, NH), 9.10 (1H, d, *J* = 1.4 Hz, H3), 8.55 (1H, d, *J* = 1.4 Hz, H6), 3.89 (2H, s, SCH_2_), 2.78 (2H, t, *J* = 7.3 Hz, CH_2_), 2.37 (3H, s, CH_3_), 1.79–1.60 (2H, m, CH_2_), 1.38–1.22 (4H, m, CH_2_), 0.84 (3H, t, *J* = 7.3 Hz, CH_3_); ^13^C-NMR (75 MHz, DMSO-*d*_6_): δ 174.2, 166.7, 160.2, 157.5, 148.2, 142.9, 141.5, 34.5, 33.2, 31.0, 28.6, 22.1, 14.1, 13.5; EA calculated for C_14_H_19_N_5_OS (305.40): 55.06% C; 6.27% H; 22.93% N; 10.50% S. Found 55.38% C; 6.37% H; 22.70% N; 10.04% S.

*2-{[1-(5-Hexylpyrazin-2-yl)ethylidene]hydrazono}1,3-thiazolidin-4-one* (**11f**): White solid; yield 52%; m.p. 162–163 °C; IR (ATR-Ge): 3124, 3071 (NH), 2997, 2975, 2946, 2922, 2849, 2814 (CH), 1704 (C=O), 1623 (C=N) cm^−1^; ^1^H-NMR (300 MHz, DMSO-*d*_6_): δ 12.11 (1H, bs, NH), 9.10 (1H, d, *J* = 1.1 Hz, H3), 8.54 (1H, d, *J* = 1.1 Hz, H6), 3.89 (2H, s, SCH_2_), 2.78 (2H, t, *J* = 7.3 Hz, CH_2_), 2.37 (3H, s, CH_3_), 1.75–1.58 (2H, m, CH_2_), 1.35–1.18 (6H, m, CH_2_), 0.83 (3H, t, *J* = 7.3 Hz, CH_3_); ^13^C-NMR (75 MHz, DMSO-*d*_6_): δ 174.2, 166.7, 160.1, 157.4, 148.2, 142.9, 141.5, 34.5, 33.2, 31.2, 28.9, 28.5, 22.2, 14.1, 13.4; EA calculated for C_15_H_21_N_5_OS (319.43): 55.40% C; 6.63% H; 21.93% N; 10.04% S. Found 55.96% C; 6.59% H; 21.92% N; 9.43% S.

*2-{[1-(5-Benzylpyrazin-2-yl)ethylidene]hydrazono}-1,3-thiazolidin-4-one* (**11g**): White solid; yield 86%; m.p. 242–243 °C; IR (ATR-Ge): 3149, 3067 (NH), 2995, 2965, 2930, 2798 (CH), 1702 (C=O), 1625 (C=N) cm^−1^; ^1^H-NMR (300 MHz, DMSO-*d*_6_): δ 12.12 (1H, bs, NH), 9.10 (1H, s, H-3), 8.63 (1H, s, H-6), 7.32–7.16 (5H, m, benzyl H-2, H-3, H-4, H-5, H-6), 4.16 (2H, s, CH_2_), 3.89 (2H, s, SCH_2_), 2.36 (3H, s, CH_3_); ^13^C-NMR (75 MHz, DMSO-*d*_6_): δ 174.2, 166.8, 160.0, 156.2, 148.5, 143.1, 141.7, 138.9, 129.2, 128.8, 126.7, 40.8, 33.2, 13.5; EA calculated for C_16_H_15_N_5_OS (325.39): 59.06% C; 4.65% H; 21.52% N; 9.85% S. Found 58.79% C; 4.81% H; 21.99% N; 10.22% S.

*2-({1-[5-(4-Methoxybenzyl)pyrazin-2-yl]ethylidene}hydrazono)-1,3-thiazolidin-4-one* (**11h**): White solid; yield 63%; mp 223–224 °C; IR (ATR-Ge): 3125, 3078 (NH), 2969, 2924, 2829 (CH), 1704 (C=O), 1627 (C=N) cm^−1^; ^1^H-NMR (300 MHz, DMSO-*d*_6_): δ 12.11 (1H, bs, NH), 9.09 (1H, s, H-3), 8.60 (1H, s, H-6), 7.26–7.14 (2H, m, AA′, BB′, benzyl H-2, H-6), 6.91–6.78 (2H, m, AA′, BB′, benzyl H-3, H-5), 4.09 (2H, s, CH_2_), 3.90 (2H, s, SCH_2_), 3.70 (3H, s, OCH_3_), 2.37 (3H, s, CH_3_); ^13^C-NMR (75 MHz, DMSO-*d*_6_): δ 174.2, 166.8, 160.0, 158.1, 156.6, 148.4, 143.0, 141.6, 130.8, 130.2, 114.2, 55.2, 33.2, 30.6, 13.4; EA calculated for C_17_H_17_N_5_O_2_S (355.42): 57.45% C; 4.82% H; 19.71% N; 9.02% S. Found 56.96% C; 4.98% H; 19.99% N; 9.40% S.

### 3.2. Biology

#### Evaluation of In Vitro Antifungal Activity

The strains were subcultured on Sabouraud dextrose agar (SDA, Difco/Becton Dickinson, Detroit, MI, USA) and maintained on the same medium at 4 °C. For susceptibility testing, fungal inocula were prepared by suspending yeasts, conidia, or sporangiospores in sterile 0.85% saline. The cell density was adjusted using a Bürker’s chamber to yield a stock suspension of 1.0 ± 0.2 × 10^5^ colony forming units (CFU)/mL and 1.0 ± 0.2 × 10^6^ CFU/mL for yeasts and molds, respectively. The final inoculum was made by 1:20 dilution of the stock suspension with the test medium. The compounds were dissolved in DMSO, and the antifungal activity was determined in RPMI 1640 media (KlinLab, Prague, Czech Republic) buffered to pH 7.0 with 0.165 M 3-morpholinopropane-1-sulfonic acid (Sigma-Aldrich, St. Louis, MO, USA). Controls consisted of medium and DMSO alone. The final concentration of DMSO in the test medium did not exceed 1% (*v/v*) of the total solution. The minimum inhibitory concentration , was defined as 80% or greater (for yeasts and yeast-like organisms—IC_80_), respectively 50% or greater (for molds—IC_50_) reduction of growth in comparison with the control. The values of MICs were determined after 24 and 48 h of static incubation at 35 °C. In the case of *T. interdigitale*, the MICs were recorded after 72 and 120 h due to its slow growth rate. Fluconazole, voriconazole, and amphotericin B were used as reference antifungal drugs. For the results, see Table 1.

## 4. Conclusions

The studied 2-{[1-(5-alkyl/arylalkylpyrazin-2-yl)ethylidene]hydrazono}-1,3-thiazolidin-4-ones showed promising activity against *C. glabrata*—an opportunistic pathogenic yeast that is often resistant to both azoles and echinocandins. The most potent derivative exhibited good activity against all eight fungal pathogens used in the susceptibility assay. In the light of the results obtained in the present study and the antifungal properties of 1,3-thiazolidin-4-one derivatives reported by other research groups, it can be concluded that substituted 1,3-thiazolidin-4-ones deserve additional studies as potential antifungal drugs.

## Figures and Tables

**Table 1:** In vitro antifungal activity of thiosemicarbazones **10g** and **10h** and 2-{[1-(5-alkyl/arylalkylpyrazin-2-yl)ethylidene]hydrazono}-1,3-thiazolidin-4-ones **11a**–**11h**.

**Compound**	**MIC (mg/L) ^1^**							
	**CA**	**CT**	**CK**	**CG**	**TA**	**AF**	**LC**	**TI**
	24 h	24 h	24 h	24 h	24 h	24 h	24 h	72 h
	48 h	48 h	48 h	48 h	48 h	48 h	48 h	120 h
**10g**	˃142.69	˃142.69	8.92	4.46	4.46	17.84	35.67	8.92
	˃142.69	˃142.69	8.92	4.46	4.46	17.84	35.67	8.92
**10h**	˃157.70	˃157.70	19.71	4.93	19.71	39.42	19.71	4.93
	˃157.70	˃157.70	19.71	4.93	19.71	39.42	19.71	4.93
**11a**	**2.17**	**2.17**	**2.17**	**1.08**	**2.17**	4.33	4.33	**2.17**
	**2.17**	**2.17**	**2.17**	**1.08**	**2.17**	8.67	4.33	**2.17**
**11b**	1.08	2.17	2.17	**1.08**	2.17	17.33	8.67	2.17
	1.08	4.33	4.33	**1.08**	2.17	17.33	17.33	2.17
**11c**	**1.14**	**1.14**	**1.14**	**0.57**	**1.14**	**2.28**	**2.28**	**1.14**
	**1.14**	**1.14**	**1.14**	**0.57**	**1.14**	**4.55**	**2.28**	**1.14**
**11d**	4.55	˃36.42	˃36.42	**2.28**	2.28	˃36.42	9.11	9.11
	9.11	˃36.42	˃36.42	**2.28**	2.28	˃36.42	9.11	9.11
**11e**	**2.39**	**2.39**	**2.39**	**2.39**	**2.39**	**2.39**	**2.39**	**2.39**
	**2.39**	**2.39**	**2.39**	**2.39**	**2.39**	**2.39**	**9.54**	**2.39**
**11f**	9.98	˃39.93	˃39.93	**1.25**	2.49	39.93	39.93	˃39.93
	9.98	˃39.93	˃39.93	**1.25**	2.49	˃39.93	39.93	˃39.93
**11g**	2.54	5.08	5.08	**2.54**	5.08	˃40.67	˃40.67	10.17
	5.08	10.17	5.08	**2.54**	5.08	˃40.67	˃40.67	20.34
**11h**	2.78	5.55	5.55	**2.78**	2.78	22.21	11.11	5.55
	2.78	11.11	5.55	**2.78**	2.78	44.43	22.21	5.55
**FLU**	0.07	˃153.14	38.29	12.76	76.57	˃153.14	˃153.14	1.99
	0.07	˃153.14	76.57	76.57	76.57	˃153.14	˃153.14	31.85
**VOR**	0.002	43.67	0.23	29.2	1.14	0.17	72.66	0.03
	0.002	87.33	0.68	87.33	5.00	0.45	87.33	0.04
**AmpB**	0.03	0.08	0.13	0.03	1.00	0.17	1.00	1.00
	0.06	0.10	0.17	0.08	1.66	0.21	2.00	1.00

^1^ Minimum inhibitory concentration (MIC) = 80% or greater for yeasts and yeast-like organisms (IC_80_), respectively 50% or greater for molds (IC_50_); CA *= Candida albicans* ATCC 44859, CT = *Candida tropicalis* 156, CK = *Candida krusei* E 28, CG = *Candida glabrata* 20/I, TA = *Trichosporon asahii* 1188, AF = *Aspergillus fumigatus* 231, LC = *Lichtheimia corymbifera* (formerly *Absidia corymbifera*) 272, and TI = *Trichophyton interdigitale* (formerly *T. mentagrophytes*) 445; FLU = fluconazole, VOR = voriconazole, AmpB = amphotericin B.
